# Evaluating physical functioning in critical care: considerations for clinical practice and research

**DOI:** 10.1186/s13054-017-1827-6

**Published:** 2017-10-04

**Authors:** Selina M. Parry, Minxuan Huang, Dale M. Needham

**Affiliations:** 1 Department of Physiotherapy, School of Health Sciences, The University of Melbourne, Melbourne, Victoria Australia; 2 Outcomes after Critical Illness and Surgery Group, Johns Hopkins University, Baltimore, MD USA; 3 Division of Pulmonary and Critical Care Medicine, School of Medicine, Johns Hopkins University, Baltimore, MD USA; 4 Department of Physical Medicine and Rehabilitation, School of Medicine, Johns Hopkins University, Baltimore, MD USA; 5 Johns Hopkins University, 1830 East Monument Street, 5th Floor, Baltimore, MD 21205 USA

**Keywords:** Critical illness, Physical function, Outcome measurement, Early mobility, Physical rehabilitation

## Abstract

The evaluation of physical functioning is valuable in the intensive care unit (ICU) to help inform patient recovery after critical illness, to identify patients who may require rehabilitation interventions, and to monitor responsiveness to such interventions. This viewpoint article discusses: (1) the concept of physical functioning with reference to the World Health Organization International Classification of Functioning, Disability and Health; (2) the importance of measuring physical functioning in the ICU; and (3) methods for evaluating physical functioning in the ICU. Recommendations for clinical practice and research are made, along with discussion of future directions.

**Electronic supplementary material:**

The online version of this article (doi:10.1186/s13054-017-1827-6) contains supplementary material, which is available to authorized users.

## Introduction

Improving the survivorship experience of patients is a defining challenge for modern critical care medicine due to improving mortality and increasing awareness of patient morbidity [1–3]. Intensive care unit (ICU) survivors with multi-organ failure are particularly susceptible to physical morbidity, with up to 30% muscle loss within the first 10 days of ICU admission [4, 5]. The prevalence of ICU-acquired weakness is 25–40% in patients ventilated for ≥ 48 h [6–8] and even higher in patients with sepsis or a prolonged ICU length of stay (LOS) [9–11]. Importantly, weakness and physical functioning are predictive of subsequent LOS, post-discharge survival, healthcare utilization, quality of life (QOL), and return to home [12–14]. The evaluation of physical functioning in the ICU is needed to help inform patient recovery, identify patients who may require rehabilitation interventions, and monitor intervention responsiveness.

### Physical functioning in the context of the International Classification of Functioning (ICF) framework

The World Health Organization (WHO) ICF framework defines functioning as an umbrella term for the interaction between three distinct constructs: body function and structure (physiological and anatomical structure of the body systems), activities (execution of a specific task within a standardized environment), and participation (involvement in everyday life situations) [15]. The ICF framework explicitly recognizes that functioning is affected by the interplay between an individual’s health condition and contextual factors, which may include personal (e.g., education) and environmental/social (e.g., home set-up, family support) factors [15].

Using this framework, physical functioning can be evaluated across the three ICF constructs. First, functioning can be evaluated in terms of physiological impairment at the level of individual organs or body systems (i.e., the “body function” level of assessment of the ICF) [15], with a specific focus on the neurological, cardiac, respiratory, and musculoskeletal systems. Second, functioning can be evaluated in terms of performance-based measurement focused on limitations in specific activities, such as sitting, standing, or walking [15]. Third, evaluation can include assessment of participation restrictions, such as the ability to perform activities of daily living (ADLs). These perspectives evaluate distinct aspects of physical functioning and thus structure and function impairment (e.g., muscle weakness) does not necessarily strongly correlate with activity limitations (e.g., 6-min walk test) and participation restrictions (e.g., ADLs) [13, 16, 17].

### Importance of measuring physical functioning in the ICU

While post-ICU impairments in physical functioning are common, our understanding of the specific subgroups of patients at highest risk for such impairments, and with the greatest potential benefit from rehabilitation interventions, is evolving. Measuring physical functioning early and longitudinally in the ICU is important to identify patients at risk of poor physical outcomes, monitor intervention efficacy, and inform recovery trajectories [12, 18, 19].

**Fig. 1:**
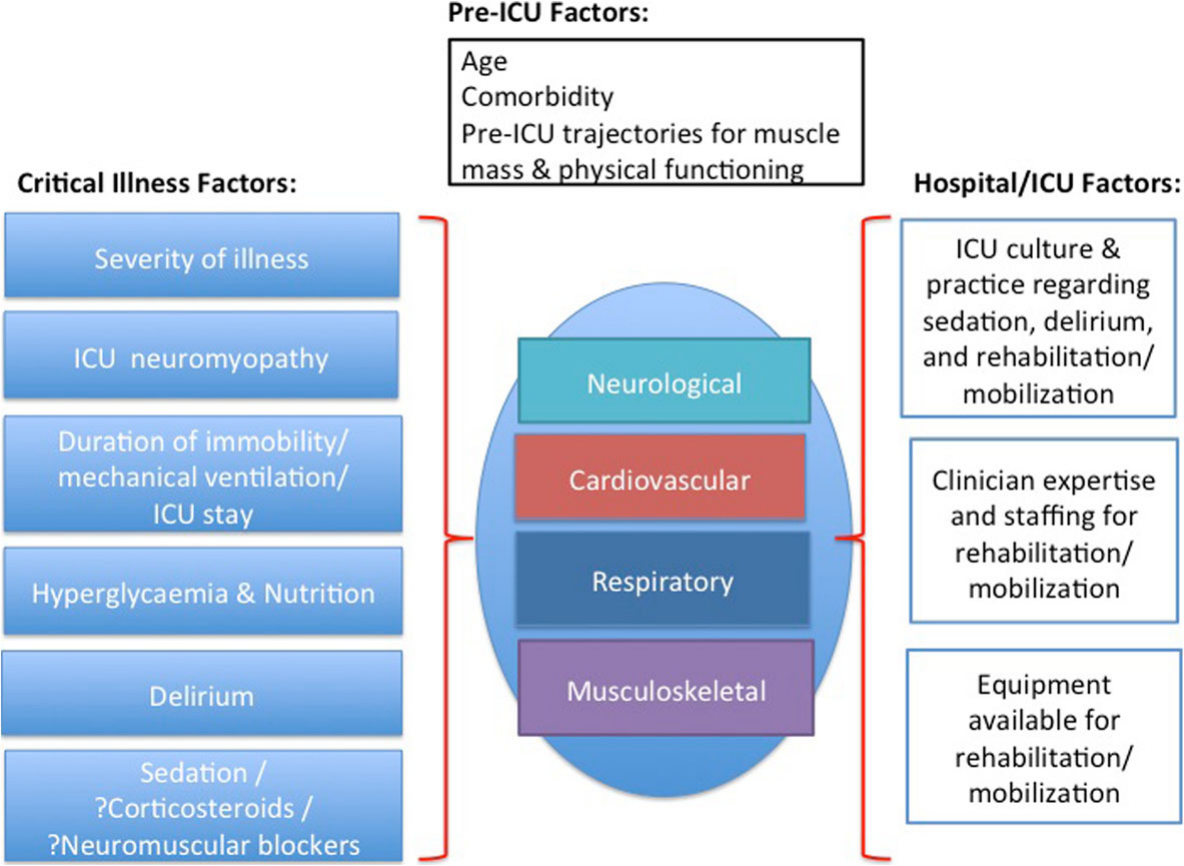
Impact of pre-ICU, critical illness and hospital/ICU factors on body systems related to physical functioning. Pre-ICU, critical illness, environmental factors, and body-system impairments, have interdependent effects on physical functioning (e.g., ICU culture regarding sedation may lead to neurological impairment resulting in immobility and musculoskeletal impairment)

Pre-ICU factors, such as age, comorbidities, and pre-ICU trajectories for muscle mass and physical functioning, impact on the physical functioning of patients in the ICU (Fig. 1). In addition, there are many factors related to critical illness and the ICU environment that can impact on impairment in physiological body systems that are critical to the physical functioning of patients in the ICU (Fig. 1).

## Major considerations in choosing an instrument

**Fig. 2:**
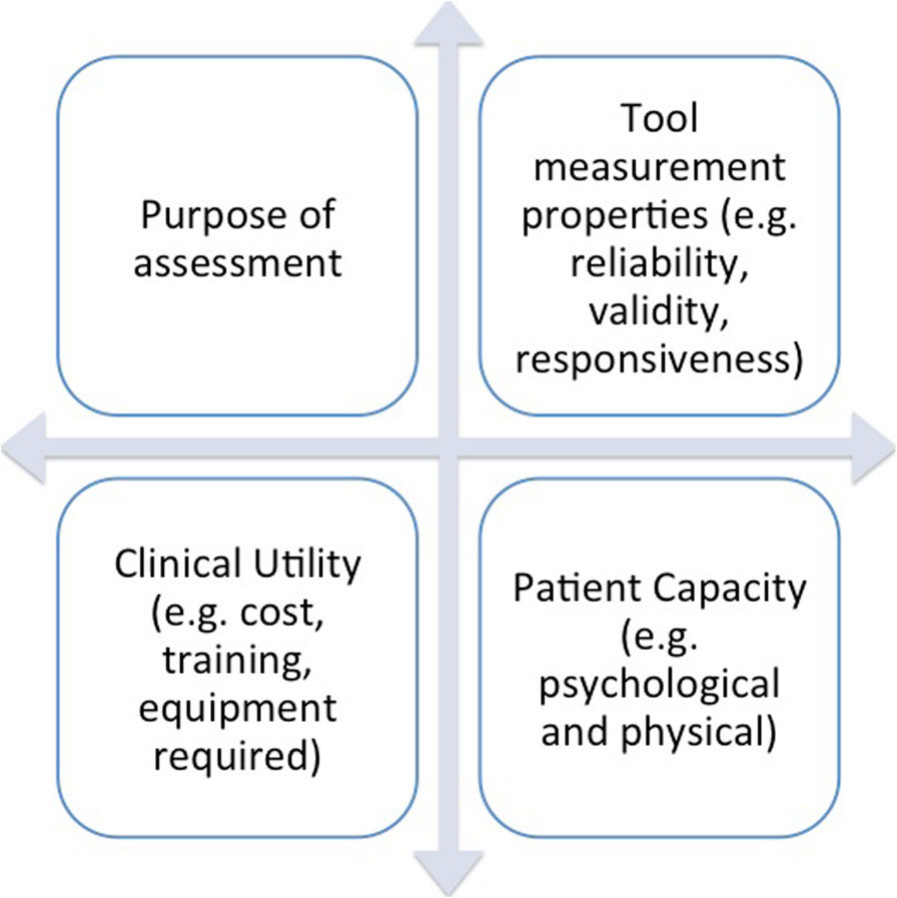
Factors to consider when selecting an outcome measure

In this next section we discuss four major considerations when selecting an instrument (Fig. 2) and synthesise current evidence (Table 1; Additional file 1: Table S1).

### Purpose of assessment

The evaluation of physical functioning is complex and is influenced by multiple interacting factors, including strength, range of motion, proprioception, balance, cognition, and psychological issues (e.g., motivation) [20]. There are also unique patient and environmental factors (e.g., sedation, severity of illness, medical devices) specific to the ICU. Determining the specific purpose for assessing physical functioning is important when selecting an appropriate instrument. For example, if the purpose is to evaluate intervention efficacy, users should consider the specific effect of the intervention and match it with an instrument that evaluates that effect. Table 3 highlights that there are important differences when each physical function instrument is mapped to the relevant subdomains of the ICF framework. For example, the Chelsea Critical Care Physical Assessment Tool assesses both respiratory and mobility ICF subdomains; the ICU Mobility Scale only evaluates mobility subdomains; and the Physical Functional in ICU Test-scored is a composite measure of mobility, strength, and endurance. Hence, if the primary aim of an intervention is to improve patient mobility via increased muscle strength, it may be most appropriate to use a composite instrument which evaluates mobility and strength (e.g., Physical Functional in ICU Test-scored or Chelsea Critical Care Physical Assessment Tool) or separate instruments individually focused on strength and mobility (e.g., dynamometry, plus ICU Mobility Scale or Functional Status Score for the ICU). Whilst domains such as climbing and jumping, which are evaluated within the Acute Care Index of Function and the Critical Care Functional Rehabilitation Outcome Measure, are less relevant during an ICU admission, they are relevant later in the recovery process. Assessment of ‘climbing’ or stair walking ability is often a critical consideration in evaluating a patient’s safety for discharge to home. Currently there is not a single measure available that can be utilized across the entire recovery trajectory. Therefore, consideration of the elements evaluated under the subdomains of the ICF framework is important when selecting the relevant instrument based on the assessment purpose.

### Measurement properties

Relevant measurement properties to consider when selecting an instrument include the ability to measure what is intended (validity). This includes subjective interpretation (face validity), whether the instrument’s content adequately reflects the parameter of interest (content validity), comparison with other tools measuring a similar construct (construct validity), and prediction of future outcomes (predictive validity) [21, 22]. In addition, the ability to obtain accurate results within or between assessors (intra- and inter-rater reliability, respectively), or when measures are repeated longitudinally (test-retest reliability) is important. Instruments should detect change over time (responsiveness) and have a limited floor (proportion of patients scoring the lowest score possible) and ceiling (proportion of patients scoring the highest score possible) effect across the expected evaluation time points [22].

Notably, caution is needed if trying to extrapolate instruments developed for one setting or patient population to the ICU setting. This issue is particularly important given many unique issues within the ICU, including sedation, delirium, fatigability, and weakness, that can affect patient performance. For example, Acute Care Index of Function, De Morton Mobility Index, and Short Physical Performance Battery were initially developed for use in non-ICU patient populations (e.g., geriatrics and neurology), [23–25] with relatively little evaluation, at present, within the ICU setting.

Table 1 and Additional file 1 (Table S1) synthesize data on measurement properties for each instrument. An adequate level of inter-rater reliability has been demonstrated for all measurement instruments, except the Short Physical Performance Battery, although this instrument has established reliability in geriatrics. All instruments have evidence of construct validity compared to other concurrent measurements of physical function and/or strength, except the Critical Care Functional Rehabilitation Outcome Measure. This instrument only has published data on face/content validity. Seven measurement instruments have evidence of predictive validity. The most commonly evaluated predictive outcome was discharge to home, which was evaluated in six instruments. The Short Physical Performance Battery was the only instrument not predictive of discharge to home, albeit the study may have been under-powered for this assessment [26]. Accurately predicting individuals unable to be discharged to home is important to help optimize the consistency, appropriateness, and timeliness of discharge planning recommendations and rehabilitation referral. There has been limited evaluation of the predictive validity beyond hospital discharge with only three instruments (ICU Mobility scale, Surgical Optimal Mobility scale, and Physical Functional in ICU Test-scored) examining post-hospital mortality with conflicting findings (Table 1; Additional file 1: Table S1).

**Table 1:** Summary of measurement properties of physical functioning instruments for the ICU

Instrument name (range for score)	Evidence of reliability?	Evidence of validity?	Evidence of predictive validity?	Evidence of responsiveness?	Evidence for MID?	Evaluation of floor and ceiling effects?^#^
ACIF (0–1)	Yes	Construct validity: Yes	Yes: for discharge to home	No	No	Low floor and ceiling in ICU
CPAx (0–50)	Yes	Content validity: Yes Construct validity: Yes	Yes: for discharge to home	Yes^a^	Yes^a^	High floor at ICU admission; Low floor and ceiling at ICU and hospital discharge^a^
CcFROM (0–63)	Yes	Face/content validity: Yes	No	No	No	Low floor and ceiling in ICU
DEMMI (0–100)	Yes	Convergent validity: Yes Divergent validity: Yes	No	No	No	Low floor and ceiling in ICU
FSS-ICU (0–35)	Yes	Construct validity: Yes Discriminant validity: Yes Known groups validity: Yes	Yes: for discharge to home and post-ICU hospital LOS^b^	Yes	Yes	Low floor and ceiling at awakening and ICU discharge, high ceiling at hospital discharge
IMS (0–10)	Yes	Construct validity: Yes Divergent validity: Yes	Yes: for discharge to home and 90-day survival^b^	Yes	No	High floor at ICU admission; Low floor and ceiling at ICU awakening and ICU discharge
MMS (0–7)	Yes	Construct validity: Yes	Yes: for post-ICU hospital LOS	No	No	High floor during ICU stay
Perme (0–32)	Yes	Construct validity: Yes	No	No	No	High floor during ICU stay
PFIT-s (0–10)	Yes	Construct validity: Yes Divergent validity: Yes	Yes: for discharge to home, post-ICU hospital LOS; Not predictive of 28-day and 12-month mortality^c^	Yes	Yes	High floor at ICU admission; Low floor and ceiling at awakening and ICU discharge
SOMS (0–4)	Yes	Construct validity: Yes Divergent validity: Yes	Yes: for ICU and hospital LOS, and in-hospital mortality^d^	No	No	Low floor and ceiling at ICU admission
SPPB (0–12)	No	Construct validity: Yes Divergent validity: Yes	Not predictive of discharge to home^b^	Yes	Yes	High floor at awakening and ICU discharge

^#^A low floor and ceiling effect is necessary. A low floor/ceiling effect was defined as <15%, and high floor/ceiling effect as >15% at any time point [26]

^a^The MID has only been reported within the burns population for the CPAx; floor and ceiling effects have mainly been reported for the burns population. At ICU discharge the floor and ceiling effect was 13% and 0% in the burns population versus a floor and ceiling effect of 3% and 1% in a general ICU population

^b^Predictive validity for FSS-ICU, IMS, and SPPB were evaluated from ICU discharge physical functioning scores

^c^Predictive validity for PFIT-s were evaluated from ICU admission (scores evaluated a median of 6 days (range 5–9 days) after admission for all patient outcomes except discharge to home which has been evaluated across three time points: ICU admission, ICU awakening, and ICU discharge)

^d^Predictive validity for SOMS was evaluated from baseline ICU admission scores

*ACIF* Acute Care Index of Function, *CPAx* Chelsea Critical Care Physical Assessment Tool, *CcFROM* Critical Care Functional Rehabilitation Outcome Measure, *DEMMI* De Morton Mobility Index, *FSS-ICU* Functional Status Score for the ICU, *ICU* intensive care unit, *IMS* ICU mobility scale, *LOS* length of stay, *MID* minimal important difference, *MMS*, *Perme* Perme ICU Mobility Score, *PFIT-s* Physical Function in intensive care test scored, *SOMS* Surgical Optimal Mobility Scale, *SPPB* Short Physical Performance Battery, *MMS* Manchester Mobility Score

The ability to detect a clinically meaningful change over time (responsiveness) was examined in five instruments (Table 1; Additional file 1: Table S1): the Chelsea Critical Care Physical Assessment Tool; the Functional Status Score for the ICU; the ICU Mobility Scale; the Physical Functional in ICU Test-scored; and the Short Physical Performance Battery. All demonstrated significant change over time within the ICU, and moderate to large effect sizes (an indicator of moderate to good responsiveness) were observed for the Functional Status Score for the ICU and Physical Functional in ICU Test-scored.

The presence of floor and ceiling effects are important considerations in assessing the recovery trajectories of patients and the intervention efficacy [22]. High floor or ceiling effects indicate that the instrument is too challenging or too easy, respectively, limiting its ability to detect a change in the physical functioning of patients. The majority of instruments have low floor and ceiling effects during an ICU stay (Table 1; Additional file 1: Table S1). However, the Short Physical Performance Battery demonstrated large floor effects which limits its potential utility in the ICU (Table 1; Additional file 1: Table S1).

Based on published measurement properties alone, the most robust ICU instruments are: Physical Functional in ICU Test-scored; Chelsea Critical Care Physical Assessment Tool; Functional Status Score for the ICU; and ICU Mobility Scale (Table 1). Ongoing research is needed to further understand the measurement properties of existing instruments to ensure appropriateness and usability within the ICU setting.

### Patient capacity

**Fig. 3:**
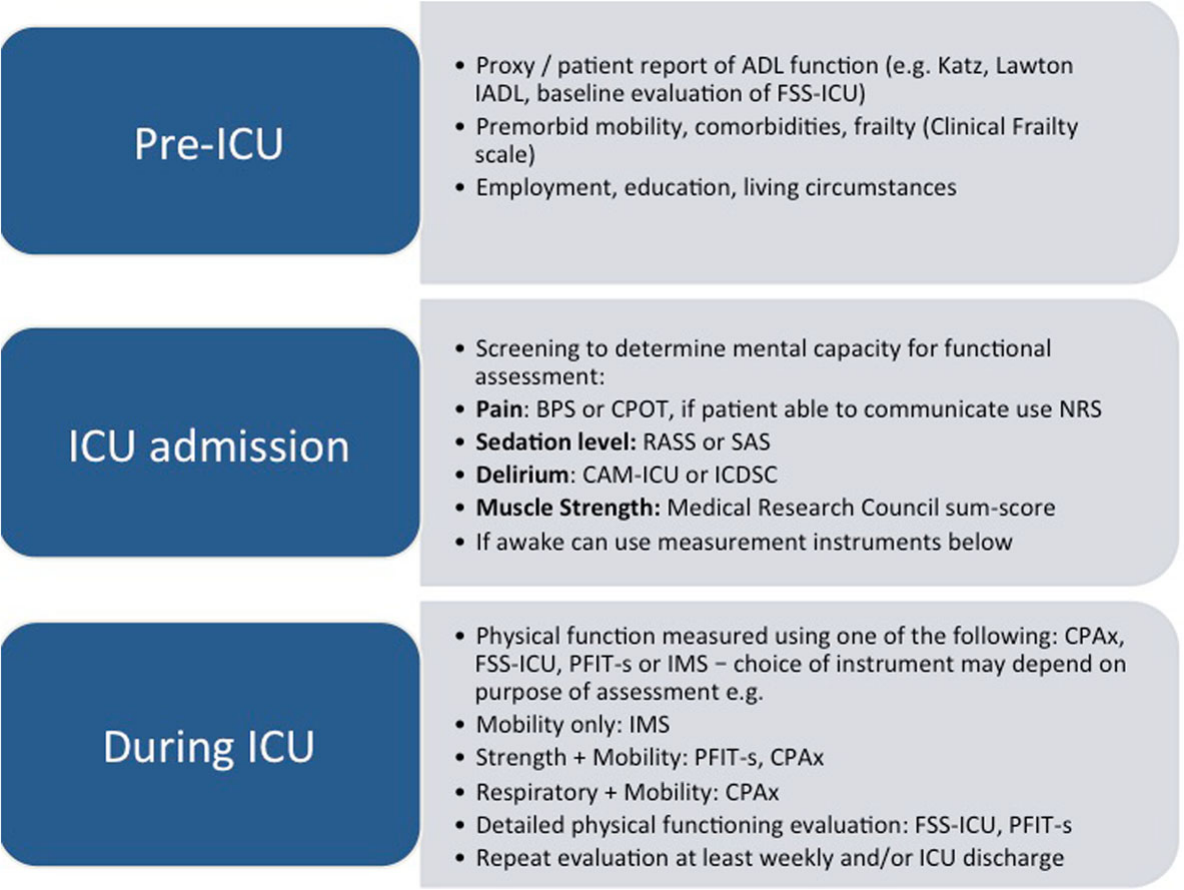
Recommendations for Clinical Practice – Measurement of Physical Functioning. Abbreviations: *ADL* activities of daily living; *BPS* Behavioural Pain Scale; *CAM-ICU* Confusion assessment method for the ICU; *CPAx* Chelsea Physical assessment Tool; *CPOT* Critical Care Pain Observation Tool; *FSS-ICU* Functional Status Score for the ICU; *IADL* instrumented activities of daily living; *ICU* intensive care unit; *ICDSC* Intensive Care Delirium Screening Checklist; *IMS* ICU Mobility Scale; *NRS* Numerical rating scale; *PFIT-s* Physical Function in ICU Test-scored; *RASS* Richmond Agitation and Sedation Scale; *SAS*, Sedation Agitation Scale

All instruments outlined herein (Tables 1 and 2) are dependent on patient effort. Consequently, assessing the feasibility of each instrument’s use within the ICU is critical. Feasibility should consider the requirements of the instrument, including issues related to a patient’s alertness, ability to follow instructions, motivation, weakness, and fatigability. A standardized method for determining patient mental capacity (including validated and reliable determinations of pain, sedation, and delirium status) is important to enable comparison of results across patients [27] (Fig. 3). The Perme ICU Mobility Score is unique as it includes evaluation of potential barriers to mobility that may affect patient performance (e.g., medical devices, pain, and respiratory support). Impairments in the balance of patients may also affect performance, with the De Morton Mobility Index and Short Physical Performance Battery including balance evaluation.

**Table 2:** Clinical utility and practical considerations of functional measures in the ICU setting

Outcome measure	Type of assessment^#^	Patient population with original development	Equipment required*	Scoring information (minimum to maximum score)	Time required to physically undertake testing	Training Rresources
ACIF [32]	Comprehensive	Acute neurological (including neurosurgery) [23]	Access to 5 stairs; walking marker (distance)	20 items, 4 subcategories (0–1.00)	12 min	No specified training package or video currently available, instructions and recording sheet available [23]
CPAx [33, 34–36]	Comprehensive	General and trauma ICU [31]	Handgrip dynamometer^a^	10 items, each scored 0–5 (0 –50)	2–10 min	Online 40–60 min free training (requires registration) at http://cpax.ocbmedia.com
CcFROM [37]	Comprehensive	General, neurosurgery and trauma ICU [33]	Stopwatch	9 items, each scored 0–7 (0–63)	10–30 min	Instructions and recording sheet available [33], no training package or video currently available
DEMMI [38]	Comprehensive	General hospitalized geriatric medical patients [24]	Chair with 45 cm seat height with arm rests; stopwatch; pen (DEMMI item 13); walking marker (distance)	15 items, 5 subcategories, each scored 0–2 (0–100)	10–30 min	Instructions and recording sheet available in supplementary [24] (no details specific to the ICU setting)
FSS-ICU [26, 31, 39, 40–42]	Comprehensive	Medical ICU [37]	Walking marker (distance)	5 items, each scored 0–7 (0–35)	10–30 min	Detailed free instructions (registration required) at www.improvelto.com/, free training package including video available from primary author^1^
IMS [43, 26, 44, 45]	Simple	General ICU (medical, surgical, trauma) [28]	None	1 item, score based on highest classification level (11 options) (0–10)	<1 min	Instructions available [28]
MMS [46]	Simple	General ICU (medical, surgical, trauma) [39]	None	1 item, score based on highest classification level (7 options) (0–7)	<1 min	Instructions and recording sheet available [39]—further detailed instructions available from primary author^2^, no training package or video currently available
PFIT-s [26, 30, 47, 48]	Comprehensive	General ICU (medical, surgical) [43]	Stopwatch; Borg RPE sheet (optional)	4 items, individual items scored 0–3 (0–10)	10–15 min	Free training package, including video, available from primary author^3^
Perme Score [49, 50, 44]	Comprehensive	General ICU; cardiovascular ICU [46, 47]	None	15 items, individual items scored 0–3 (0–32)	15–60 min	No training package or video currently available. The scoring criteria and detailed instructions are available in the manuscript [46]
SOMS [51, 52–54]	Simple	Surgical ICU [49]	None	1 item, score based on highest classification level (5 options) (0–4)	<1 min	No training package or video currently available, scoring criteria available in manuscript [49]
SPPB [26]	Comprehensive	Geriatric, non-hospitalized	Stopwatch; tape-measure (for 4-m course)	3 items, each item scored 0–4 (0–12)	5–10 min	Free training via: https://www.irp.nia.nih.gov/branches/leps/sppb/index.htm and https://www.youtube.com/watch?v=XgiuciJXPm4 (no details specific for ICU)

*Additional equipment required beyond standard hospital bed, chair, and gait aids

^#^Type of assessment was defined into two categories: 1) “simple” involving observation of patient’s current ability (time to complete: <5 min); and 2) “Comprehensive” providing greater understanding of the impairments in physical functioning (time to complete: 10–15 min)

^1^Dale Needham, School of Medicine, Johns Hopkins University. Contact email: dale.needham@jhmi.edu

^2^David Williams, Therapy Services, University Hospitals Birmingham NHS Foundation Trust. Contact email: david.mcwilliams@uhb.nhs.uk

^3^Linda Denehy, Physiotherapy Department, The University of Melbourne. Contact email: l.denehy@unimelb.edu.au

^a^ A table is able to be downloaded at the end of the eLearning module which provides the gender/age values for handgrip strength in order to work out percentage grip strength which is required to complete the CPAx

*ACIF* Acute Care Index of Function, *CPAx* Chelsea Critical Care Physical Assessment Tool, *CcFROM* Critical Care Functional Rehabilitation Outcome Measure, *DEMMI* De Morton Mobility Index, *FSS-ICU* Functional Status Score for the ICU, *ICU* intensive care unit, *IMS* ICU mobility scale, *MMS*, *Perme* Perme ICU Mobility Score, *PFIT-s* Physical Function in intensive care test scored, *RPE* rating of perceived exertion, *SOMS* Surgical Optimal Mobility Scale, *SPPB* Short Physical Performance Battery, *MMS* Manchester Mobility Score

### Clinical utility

The levels of expertise, training, and time required, as well as any specialized equipment, are important in assessing clinical utility. All instruments require minimal additional equipment, apart from the Chelsea Critical Care Physical Assessment Tool which requires a handgrip dynamometer, and the Acute Care Index of Function which requires a set of five steps (Table 2). Dedicated ICU training packages are available for three instruments: Chelsea Critical Care Physical Assessment Tool; Functional Status Score for the ICU; and Physical Function in ICU test-scored (Table 2). The fastest tests are the simple one-item mobility scales that indicate the patients highest level, while other more comprehensive instruments require more time to assess multiple specific activities and/or levels of assistance required (Table 2).

### Recommendations for clinical practice

We propose a staged approach for assessing physical functioning in the ICU (Fig. 3). In terms of pre-ICU status, we recommend obtaining physical functioning data as part of the patient history to inform appropriate patient goals for recovery and rehabilitation [2]. The ability to obtain a validated baseline measure of physical functioning (or pre-ICU health status) is challenging due to the severity of illness, sedation, and reduced ability of patients to engage in volitional assessments. The Clinical Frailty Scale can be used to obtain a baseline assessment of frailty. Patients who are frail prior to ICU admission have worse mortality and morbidity, and require institutionalization at discharge; thus, frailty may be a useful prognostic tool [28]. Similar to the process used with the Functional Independence Measure instrument, commonly used throughout the inpatient rehabilitation setting, it is possible to conduct a baseline assessment for the Functional Status Score for the ICU measure via proxy assessment, as performed in prior research [29]; however, this baseline version of the Functional Status Score for the ICU has not been specifically validated. The ability to measure pre-ICU physical functioning is an area for future research.

Screening for mental capacity should commence from ICU admission and include assessments of pain, sedation, and delirium status [27]. We also recommend regular screening for muscle weakness using the Medical Research Council sum-score. It is likely less important to evaluate physical functioning in ICU patients who lack muscle weakness; however, strength should not be a sole guide for determining the need for physical functioning assessment because strength and function are only weakly correlated in ICU survivors [16]. At present, there is a lack of robust, validated predictive models for physical functioning impairments within the ICU. There is a predictive model for physical functioning after hospital discharge, but not whilst in hospital [12]. Therefore, identification of patients who need evaluation of physical functioning in the ICU is largely reliant on clinical judgment regarding many potential risk factors (Fig. 1). Once the patient can follow commands, we recommend, at a minimum, one of the four recommended physical functioning tools: Physical Functional in ICU Test-scored; Chelsea Critical Care Physical Assessment Tool; Functional Status Score for the ICU; and ICU Mobility scale. Summary information about these instruments (including how to access and use them) is available through a free website: www.improveLTO.com.

When selecting specific instrument(s) for a particular ICU setting, the following are important considerations: available clinician resources and expertise; and rationale for assessment (e.g., simple versus comprehensive evaluation). In settings with limited access to rehabilitation clinicians, a simple one-item scale (e.g., ICU mobility scale) can be used, which can be feasibly completed by the bedside ICU nurse. For patients with identified mobility restrictions, consultation with physiotherapists and occupational therapists may be warranted, with more comprehensive instruments used as part of their routine clinical evaluation (Fig. 3).

## Areas for future investigation

There is an ongoing need to examine the measurement properties and clinical utility of ICU physical functioning instruments. In addition to primary measurement studies, valuable insights could be achieved through secondary analyses of existing studies that include relevant instruments, enabling larger sample sizes across multi-center trials [26, 30, 31]. Predictive validity is a critical consideration and needs additional evaluation for all instruments to assist with meaningful interpretation of the scores and the effects of associated interventions. The purpose of assessment should be considered when selecting an instrument. As highlighted in Table 3, there is variability in the subdomains evaluated across instruments. Future research is required to determine the most critical subdomains of physical functioning that always should be encompassed within evaluations in the ICU and across the recovery trajectory. It is currently unknown whether a single instrument, which encompasses all relevant subdomains and has robust measurement properties, is feasible; it is likely more than one instrument may be required.

**Table 3:** Mapping of outcome measures against ICF framework

	FSS-ICU	PFIT-s	IMS	CPAx	ACIF^#^	ccFROM	DEMMI	SOMS	SPPB	MMS	Perme^#^
Body functions											
B4. Functions of cardiovascular and respiratory systems											
Respiratory functions, other specified [b4408]				X							
Additional respiratory functions [b450]				X							
General physical endurance [b4550]		X									X
B7. Neuromuscular and movement-related functions											
Mobility of joint functions [b710]								X			
Power of isolated muscles and muscle groups [b7300]		X		X		X	X				X
Power of muscle of one limb [b7301]											
Activities and participation											
D4. Mobility											
Lying down [d4100]	X		X	X	X	X	X	X		X	X
Sitting [d4103]	X	X	X	X	X	X	X		X	X	X
Standing [d4104]					X		X	X			
Maintaining a lying position [d4150]			X					X			
Maintaining a sitting position [d4153]	X		X	X	X	X	X			X	X
Maintaining a standing position [d4154]			X	X	X	X	X		X	X	X
Transferring one-self while sitting [d4200]			X	X	X	X				X	X
Fine hand use (picking up) [d4400]							X				
Jumping [d4553]							X				
Walking short distances [d4500]	X		X		X	X	X	X	X	X	X
Walking, other specified [d4508]			X^1^	X^2^		X^3^					
Climbing [d4551]					X						
Moving around using equipment [d465]					X						

In the development of this table the World Health Organization International Classification of Functioning linkages were used from http://apps.who.int/classifications/icfbrowser/, accessed May 2016. The three most relevant domains identified were: B4—Functions of cardiovascular and respiratory system; B7—Neuromuscular and Movement-Related Functions; and D4—Mobility. The final subdomain classification is identified in the first column including coding (e.g., power of isolated muscles and muscle groups is coded b7300 in the ICF browser). Subdomains under D4—Mobility of the ICF framework not considered by these functional measures include: squatting [d4101], kneeling [d4102], bending [d4106], shifting the body’s center of gravity [d4106], maintaining a squatting or kneeling position [d4151 and d4152], transferring one-self while lying [d4201], lifting and carrying objects [d430], moving objects with lower extremities [d435], hand and arm use [d445], and walking long distances, on different surfaces and around obstacles [d4501, d4502, and d4503, respectively]

^#^The tools ACIF and Perme assess additional subdomains not outlined in the table. For ACIF, these specific subdomains are: acquiring basic skills [d1550], communicating with receiving—spoken messages [d310], and communicating when receiving—body gestures [d3150]. For Perme, these specific subdomains are: communicating with receiving—spoken messages [d310], generalized pain [D2800], and consciousness functions [b110]. Additionally, Perme had subdomains which could not be mapped to the ICF framework, including: need for mechanical ventilation or non-invasive ventilation; lines and attachments, and presence of drips

^1^In the IMS this referred to the item ‘marching on the spot (at the bedside)’

^2^In the CPAX this referred to the item ‘stepping’

^3^In the ccFROM this referred to the item ‘marching on the spot’

*ACIF* Acute Care Index of Function, *CPAx* Chelsea Critical Care Physical Assessment Tool, *CcFROM* Critical Care Functional Rehabilitation Outcome Measure, *DEMMI* De Morton Mobility Index, *FSS-ICU* Functional Status Score for the ICU, *ICF* International Classification of Functioning, *ICU* intensive care unit, *IMS* ICU mobility scale, *MMS*, *Perme* Perme ICU Mobility Score, *PFIT-s* Physical Function in intensive care test scored, *RPE* rating of perceived exertion, *SOMS* Surgical Optimal Mobility Scale, *SPPB* Short Physical Performance Battery, *MMS* Manchester Mobility Score

There is often a delay in initiating evaluations of physical functioning in the ICU due to sedation, delirium, and illness severity impacting the volitional ability of patients. Hence, during this very early stage of critical illness, non-volitional instruments may be appropriate (e.g., screening neuromuscular electrophysiological or ultrasound tests [2]). Generally, these non-volitional assessments are not part of routine clinical practice. Further examination of their clinical utility and measurement properties is needed. Future work should also explore how psychological and cognitive capacity impact patient performance, engagement, and the timing and frequency of evaluation of physical functioning.

## Conclusions

Impairment in physical functioning among ICU survivors results in significant morbidity and burden to patients, caregivers, and society. With a growing population of ICU survivors, greater utilization and standardization of physical functioning instruments is needed. This article has provided a framework and recommendations for practice. Measuring physical functioning early and longitudinally in the ICU is important to determine patients at risk of poor physical outcomes, monitor intervention efficacy, and inform recovery trajectories. These insights are important to improving the outcomes of critically ill patients.

## Additional file

Additional file 1: Table S1. Detailed summary of measurement properties for the ICU setting. (DOCX 60 kb)

## Abbreviations

ADL: Activity of daily living
ICF: International Classification of Functioning
ICU: Intensive care unit
LOS: Length of stay
QOL: Quality of life
WHO: World Health Organization
