# Supplementary material for: Evaluating physical functioning in critical care: considerations for clinical practice and research
Source: Crit Care. 2017 Oct 4;21:249. doi: 10.1186/s13054-017-1827-6 (PMC5628423; doi:10.1186/s13054-017-1827-6)
Supplement: Additional file 1: Table S1. — Detailed summary of measurement properties for the ICU setting. (DOCX 60 kb) [file 13054_2017_1827_MOESM1_ESM.docx]

**Additional file 1**: Table S1**: Detailed summary of measurement properties for the ICU setting**

| **Outcome**  **Measure and score** | **Reliability** | **Selected measures of content and concurrent validity***^a^* | **Predictive validity** | **Responsiveness***^b^* | **MID** | **Time Point with Floor / Ceiling Effects** |
| --- | --- | --- | --- | --- | --- | --- |
| **ACIF**  **(0-1)** | Inter-rater: ICC=0·4  (individual ICC range=0·81-0·94)[[1](#_ENREF_1)] | Construct validity: IMS (r=0·84)[[1](#_ENREF_1)] | DC home (ROC=0·79)[[1](#_ENREF_1)] | NR | NR | During ICU: 0% / 10%[[1](#_ENREF_1)] |
| **CPAx**  **(0-50)** | Inter-rater: ICC=0·97-1·00;  internal consistency: a=0.80[[2](#_ENREF_2), [3](#_ENREF_3)] | Content validity: s-CVI = 0·94-1·00 (percentages of items rated 3 or 4)[[2](#_ENREF_2), [3](#_ENREF_3)]  Construct validity: MRC total score (biceps/quadriceps) (r=0·65), GCS (r=0·76), SF-36 PCS (r=0·72-0·84) [[3](#_ENREF_3), [4](#_ENREF_4)] | DC home (p<0·0001) | Significant change in scores across in-patient time points^c^ (p<0.001)[[5](#_ENREF_5)] | 6·0[[2](#_ENREF_2)] | Pre-ICU: 0% / 87%[[5](#_ENREF_5)]^c^  ICU admit: 67% / 0%[[5](#_ENREF_5)] ^c^  ICU DC: 3-13%^c^ / 0-1%[[4](#_ENREF_4), [5](#_ENREF_5)]  Hospital DC: 0% / 0%[[5](#_ENREF_5)] ^c^ |
| **CcFROM**  **(0-63)** | Inter-rater: ICC=0·91  Intra-rater: ICC= 0·99 [[6](#_ENREF_6)] | Face and content validity demonstrated [[6](#_ENREF_6)] | NR | NR | NR | In ICU: Floor effect present (% NR) / 0% [[6](#_ENREF_6)] |
| **DEMMI**  **(0-100)** | Inter-rater: ICC=0·93 during ICU; after ICU DC: ICC=0·97;  Intra-rater: ICC=0·68 during ICU [[7](#_ENREF_7)] | Convergent validity: BI (r=0·56), Katz-ADL (r=-0·45), MRC-SS (r=0·57);  Divergent validity: APACHE III (r=-0·18) [[7](#_ENREF_7)] | NR | NR | NR | During ICU: 3% / 0%;  After ICU DC: 0% / 3%[[7](#_ENREF_7)] |
| **FSS-ICU**  **(0-35)** | Inter-rater: ICC=0·98-1·00 [[8](#_ENREF_8)];  Internal consistency: alpha=0·78-0·95[[9](#_ENREF_9)]; Portuguese translation inter-rater: ICC=0.84-0.91[[10](#_ENREF_10)] | Construct validity: ADL (r=0·39-0·86), IADL (r=0·48-0·57), PFIT-s (r=0·85-0·87), MRC-SS (r=0·32-0·81), IMS (r=0·46-0·95);  Discriminant validity: BMI (r=-0·37-0·16), Home 0_2_ (r=-0·12-0·22) and medication (-0·24-0·30)  Known group validity: ICU-AW, DC home (p<0·01) [[9](#_ENREF_9), [11](#_ENREF_11), [12](#_ENREF_12)] | DC home (OR=1·11, p<0·01; AUC=0·75)  Post-ICU hosp LOS (p<0·01)[[9](#_ENREF_9), [11](#_ENREF_11), [12](#_ENREF_12)] [[8](#_ENREF_8)]^d^ | ES=2·0; significant change in scores across ICU time points [[9](#_ENREF_9), [11-13](#_ENREF_11)] | 2·0-5·0[[9](#_ENREF_9)]  4·3-5·6[[11](#_ENREF_11)] | Awakening: 3% / 0-1%;  ICU DC: 0% / 3-11%;  Hospital DC: 0% / 21%[[9](#_ENREF_9), [11](#_ENREF_11)] |
| **IMS**  **(0-10)** | Inter-rater: ICC=0.80 [[14](#_ENREF_14)]; Portugese translation inter-rater: Cronbach’s alpha=0.99 [[15](#_ENREF_15)] | Construct validity: MRC-SS (r=0·57-0·64); PFIT-s (r=0·66-0·81); Perme Score (r=0·94)  Divergent validity: weight (r=0·06), gender (r=0·34)[[11](#_ENREF_11), [15](#_ENREF_15), [16](#_ENREF_16)] | DC home (OR=1·16-1·54, p<0·03)  Survival to 90 days (OR=1·38, p=0·001)[[11](#_ENREF_11), [16](#_ENREF_16)] ^d^ | ES=0·59 & 0·80; significant change in scores across ICU time points; and up to 6 months[[11](#_ENREF_11), [16](#_ENREF_16)] | NR | Study admit: 96% / NR;  Awakening: 17% / 0%;  In-ICU: 36% / 6%;  ICU DC: 0-14% / 4-5%[[11](#_ENREF_11), [15](#_ENREF_15), [16](#_ENREF_16)] |
| **MMS**  **(0-7)** | Inter-rater: k=1 [[17](#_ENREF_17)] | Construct validity: Barthel Index (r=0·880); [[17](#_ENREF_17)] | post-ICU LOS (r=0·58) [[17](#_ENREF_17)] ^d^ | NR | NR | In-ICU: 47%/8% [[17](#_ENREF_17)] |
| **Perme Score**  **(0-32)** | Inter-rater: k=0·94; individual items k=0·0-1·0 [[18](#_ENREF_18), [19](#_ENREF_19)]  Portugese translation inter-rater individual items: Cronbach’s alpha=0.96-1.00 [[15](#_ENREF_15)] | Construct validity: IMS (r=0·94) | NR | NR | NR | In-ICU: 20%/3% [[15](#_ENREF_15)] |
| **PFIT-s**  **(0-10)** | Inter-rater: ICC=0·99-1·00[[20](#_ENREF_20)] | Construct validity: FSS-ICU (r=0·85-0·87), SPPB (r=0·70-0·86), MRC (r=0·49-0·92), IMS (r=0·66-0·81);  Divergent validity: BMI (r=-0·01)[[11](#_ENREF_11), [21](#_ENREF_21), [22](#_ENREF_22)] | DC home (OR=1·20-1·59, p<0.01);  ICU-AW (OR=1·28, p<0·001)[[11](#_ENREF_11), [21](#_ENREF_21), [22](#_ENREF_22)]  Hospital LOS ( p<0·001)  28 and 12 month mortality (p>0·05)^e^  [[11](#_ENREF_11), [21](#_ENREF_21), [22](#_ENREF_22)] | Significant change in scores across ICU time points: ES=0·59-1·14 [[11](#_ENREF_11), [21](#_ENREF_21), [22](#_ENREF_22)] | 1·0-1·5 [[11](#_ENREF_11), [21](#_ENREF_21), [22](#_ENREF_22)] | ICU admit: 22% / 0%;  Awakening: 9% / 2%;  ICU DC: 2-5% / 5-11%[[11](#_ENREF_11), [21](#_ENREF_21), [22](#_ENREF_22)] |
| **SOMS**  **(0-4)** | Inter-rater (k=0·26-0·55); agreement>80% between raters, k=0·80[[23-25](#_ENREF_23)] | Construct validity: APACHE II (r=-0·31), vasopressor (r=0·47);  Divergent validity: renal failure (r=-0·21), arterial pH (r=0·24)[[23-25](#_ENREF_23)] | ICU and hospital LOS (p<0·004 in multivariable analysis);  Mortality (p=0·001 in multivariable analysis)[[23-25](#_ENREF_23)] | NR | NR | ICU admit: 5-14% / 0-13%[[23-25](#_ENREF_23)] |
| **SPPB**  **(0-12)** | NR | Construct validity: PFIT-s (r=0·70-0·86);    Divergent validity: MRC-SS (r=0·30)[[11](#_ENREF_11)] | Not predictive of DC home (p>0·05)^d^ | Significant change in scores across ICU time points; ES=0·33 [[11](#_ENREF_11)] | 1·3-1·5 [[11](#_ENREF_11)] | Awakening: 83% / 0%;  ICU DC: 57% / 0%[[11](#_ENREF_11)] |

**Abbreviations:** ACIF, Acute Care Index of Function; admit, admission; ADL, activities of daily living; APACHE, Acute Physiology and Chronic Health Evaluation; BMI, Body Mass Index; btw, between; CPAx, Chelsea Critical Care Physical Assessment Tool; CcFROM, Critical Care Functional Rehabilitation Outcome Measure; DC, discharge; DEMMI, De Morton Mobility Index; diff, difference; ES, effect size; FSS-ICU, Functional Status Score for the ICU; GCS, Glasgow Coma Scale; hosp, hospital; IADL, instrumental activities of daily living; ICC, intraclass correlation coefficient; ICU, intensive care unit; ICU-AW, Intensive Care unit Acquired weakness; IMS, ICU mobility scale; LOS, length of stay; MID, minimal important difference; MRC-SS, Medical Research Council Sum-score; O_2_, oxygen; PCS: physical component summary; Perme Score, Perme ICU Mobility Score; PFIT-s, Physical Function in intensive care test scored; pH: potential of hydrogen; ROC, Receiver operating curve; s-CVI, scale content validity index; SF-36, Short Form Health Survey; sig, significant; SOMS, Surgical Optimal Mobility Scale; SPPB, Short Physical Performance Battery; %, percentage; >, greater than.

*^a^ Strength of validity for Spearman’s correlation: excellent (=>0.75), moderate (0.50-0.74), fair (0.25-0.50), no correlation (<0.25)[*[*26*](#_ENREF_26)*].*

*NR = (not reported) no published peer reviewed journal data established for this measurement property*

*^b^ Responsiveness was determined by the effect size, and this is calculated using the mean difference divided by pooled SD. A larger effect size reflects a greater responsiveness. Thresholds for interpretation of change were: small (0.2-0.49), moderate (0.5-0.79) and large (*≥*0.80)[*[*27*](#_ENREF_27)*,* [*28*](#_ENREF_28)*]*

*^c^ The MID has only been reported within the burns population for the CPAx, floor and ceiling effects have mainly been reported for the burns population. At ICU discharge the floor and ceiling effect was 13% and 0% in burns population versus a floor and ceiling effect of 3% and 1% in a general ICU population.*

*^d^ Predictive validity for FSS-ICU, IMS and SPPB were evaluated from ICU discharge physical functioning scores.*

*^e^ Predictive validity for PFIT-s were evaluated from ICU admission (scores evaluated a median of 6 days [5-9] after admission for all patient outcomes except discharge to home which has been evaluated across three time-points: ICU admission, ICU awakening and ICU discharge.*

*^f^ Predictive validity for SOMS were evaluated from baseline ICU admission scores.*

**Reference List:**

1. Bissett, B., M. Green, V. Marzano, S. Byrne, I. Leditschke, T. Needman, R. Boots, and J. Paratz, *Reliability and utility of the acute care index of function in intensive care patients: An observational study.* Heart and Lung, 2016. **45**: p. 10-14.

2. Corner, E., J. Handy, and S. Brett, *Elearning to facilitate the education and implementation of the chelsea critical care physical asessment: A novel measure of function in critical illness.* BMJ Open, 2016. **6**: p. e010614.

3. Corner, E., H. Wood, C. Englebretsen, A. Thomas, R. Grant, D. Nikoletou, and N. Soni, *The Chelsea critical care physical assessment tool (CPAx): Validation of an innovative new tool to measure physical morbidity in the general adult critical care population: An observational proof of concept pilot study.* Physiotherapy, 2013. **99**: p. 33-41.

4. Corner, E., N. Soni, J. Handy, and S. Brett, *Construct validity of the Chelsea Critical Care Physical Assessment tool: An observational study of recovery from critical illness.* Critical Care, 2014. **18**: p. R55.

5. Corner, E.J., L.V. Hichens, K.M. Attrill, M.P. Vizcaychipi, S.J. Brett, and J.M. Handy, *The responsiveness of the Chelsea Critical Care Physical Assessment tool in measuring functional recovery in the burns critical care population: an observational study.* Burns, 2015. **41**(2): p. 241-7.

6. Twose, P., M. Wise, and S. Enright, *Critical care functional rehabilitation outcome measure: Developing a validated measure.* Physiotherapy Theory and Practice, 2015. **31**: p. 474-482.

7. Sommers, J., T. Vredeveld, R. Lindeboom, F. Nollet, R. Engelbert, and M. van der Schaaf, *The De Morton Mobility Index is feasible, reliable and valid in critically ill patients.* Physical Therapy, 2016. **In press**.

8. Ragavan, V., K. Greenwood, and K. Bibi, *The Functional Status Score for the Intensive Care Unit Scale: Is it reliable in the Intensive Care Unit? Can it be used to determine discharge placement?* Journal of Acute Care Physical Therapy, 2016. **7**(3): p. 93-100.

9. Huang, M., K. Chan, J. Zanni, S. Parry, S. Neto, V. da Silva, M. Kho, and D. Needham, *Functional status score for the intensive care unit (FSS-ICU): An international clinimetric analysis of validity, responsiveness and minimal important difference* Critical Care Medicine, 2016. **In Press**.

10. Maldaner da Silva, V., J. Neto, G. Cipriano, M. Pinedo, D. Needham, J. Zanni, and F. Guimaraes, *Brazilian Version of the Functional Status Score for the ICU: translation and cross-cultural adaptation.* Revista brasileira de terapia intensiva, 2017. **29**(1): p. 34-38.

11. Parry, S., L. Denehy, L. Beach, S. Berney, H. Williamson, and C. Granger, *Functional outcomes in ICU - what should we be using? An observational study.* Critical Care, 2015. **19**: p. 127.

12. Thrush, A., M. Rozek, and J. Dekerlegand, *The clinical utility of the functional status score for the intensive care unit (FSS-ICU) at a long term acute care hospital: A prospective cohort study.* Physical Therapy, 2012. **92**: p. 1536-1545.

13. Zanni, J., R. Korupolu, E. Fan, P. Pradhan, K. Janjua, J. Palmer, R. Brower, and D. Needham, *Rehabilitation therapy and outcomes in acute respiratory failure: An observational pilot project.* Journal of Critical Care, 2010. **25**(254-262).

14. Hodgson, C., D. Needham, K. Haines, M. Bailey, A. Ward, M. Harrold, P. Young, J. Zanni, H. Buhr, A. Higgins, J. Presneill, and S. Berney, *Feasibility and inter-rater reliability of the ICU Mobility Scale.* Heart & Lung: The Journal of Acute and Critical Care, 2014. **43**(1): p. 19-24.

15. Kawaguchi, W., R. Nawa, T. Figueiredo, L. Martins, and R. Pires-Neto, *Perme Intensive Care Unit Mobility Score and ICU Mobility Scale: translation into Portugese and cross-cultural adaptation for use in Brazil.* Journal Brasileiro de Pneumologia, 2016. **42**(6): p. 429-434.

16. Tipping, C., M. Bailey, R. Bellomo, S. Berney, H. Buhr, L. Denehy, M. Harrold, A. Holland, A. Higgins, T. Iwashyna, D. Needham, J. Presneill, M. Saxena, E. Skinner, S. Webb, P. Young, J. Zanni, and C. Hodgson, *The ICU Mobility scale has construct and predictive validity and is responsive: A multi-centre observational study.* Annals of American Thoracic Society, 2016. **In Press**.

17. McWilliams, D., G. Atkins, J. Hodson, M. Boyers, T. Lea, and C. Snelson, *Feasibility and reliability of the Manchester Mobility Score as a measure of physical function within the intensive care unit.* ACPRC Journal, 2016. **In Press**.

18. Perme, C., R. Nawa, C. Winkelman, and F. Masud, *A tool to assess mobility status in critically ill patients: The Perme Mobility Score.* Methodist Debakey Cardiovascular Journal, 2014. **10**(1): p. 41-49.

19. Nawa, R.K., C. Lettvin, C. Winkelman, P.R.B. Evora, and C. Perme, *Initial inter-rater reliability for a novel measure of patient mobility in a Cardiovascular ICU.* Journal of critical care, 2014.

20. Skinner, E.H., S. Berney, S. Warrillow, and L. Denehy, *Development of a physical function outcome measure (PFIT) and a pilot exercise training protocol for use in intensive care.* Crit Care Resusc, 2009. **11**(2): p. 110-5.

21. Denehy, L., N. De Morton, E. Skinner, L. Edbrooke, K. Haines, S. Warrillow, and S. Berney, *A Physical Function test for use in the intensive care unit: Validity, responsiveness, and predictive utility of the physical function icu test (scored)* Physical Therapy, 2013. **93**: p. 1636-1645.

22. Nordon-Craft, A., M. Schenkman, L. Edbrooke, D. Malone, M. Moss, and L. Denehy, *The Physical Function Intensive Care Test: Implementation in survivors of critical illness.* Physical Therapy, 2014. **94**: p. 1499-1507.

23. Schaller, S., C. Stauble, M. Susemasa, M. Heim, I. Duarte, O. Mensch, R. Bogdanski, H. Lewald, M. Eikermann, and M. Blobner, *The German validation study of the surgical intensive care unit optimal mobility score.* Journal of Critical Care, 2016. **32**(201-206).

24. Kasotakis, G., U. Schmidt, D. Perry, M. Grosse-Sundrup, J. Benjamin, C. Ryan, S. Tully, R. Hirschberg, K. Waak, G. Velmahos, E. Bittner, R. Zafonte, J. Cobb, and M. Eikermann, *The surgical intensive care unit optimal mobility scale predicts mortality and length of stay.* Critical Care, 2012. **40**: p. 1122-1128.

25. Piva, S., G. Dora, C. Minelli, M. Michelini, F. Turla, S. Mazza, P. D'Ottavi, I. Moreno-Duarte, C. Sottini, M. Eikermann, and N. Latronico, *The surgical optimal mobility score predicts mortality and length of stay in an Italian population of medical, surgical and neurologic intensive care unit patients.* Journal of Critical Care, 2015. **30**: p. 1251-1257.

26. Portney, L.G. and M.P. Watkins, *Foundations of clinical research, applications to practice*. 3rd Edition ed. 2009, Conneticut: Appleton and Lange.

27. Cohen, J., *Statistical Power Analysis for the Behavioural Sciences*. 2nd ed ed. 1988, Hillsdale: Lawrence Erlbaum.

28. Husted, J., R. Cook, V. Farewell, and D. Gladman, *Methods for assessing responsiveness: A critical review and recommendations.* Journal of Clinical Epidemiology, 2000. **53**: p. 459-468.
